# PRAME promotes invasion and metastasis of salivary adenoid cystic carcinoma by regulating RARα stability via the cullin2 ubiquitin‐proteasome system

**DOI:** 10.1002/ctm2.70787

**Published:** 2026-08-27

**Authors:** Xudong Wang, Tingyao Ma, Yixin Jing, Tianyi Li, Xi Zhao, Guohan Zhao, Ran Gao, Lu Kong, Xiaohong Chen

**Affiliations:** ^1^ Department of Otolaryngology‐Head and Neck Surgery, Beijing Tongren Hospital Capital Medical University Beijing China; ^2^ NHC Key Laboratory of Human Disease Comparative Medicine Beijing Engineering Research Center for Experimental Animal Models of Human Critical Diseases Institute of Laboratory Animal Sciences Chinese Academy of Medical Sciences (CAMS) and Comparative Medicine Center, Peking Union Medical College (PUMC) Beijing China; ^3^ Department of Biochemistry and Molecular Biology Capital Medical University Beijing China

**Keywords:** cancer‐testis antigen, cullin2 ubiquitin‐proteasome system, epithelial–mesenchymal transition, RARα, salivary adenoid cystic carcinoma

## Abstract

**Background:**

All‐trans retinoic acid (ATRA) exerts its biological functions primarily through nuclear receptors, but its therapeutic efficacy in solid tumours remains limited. This study aimed to elucidate the regulatory role of the cancer‐testis antigen PRAME in posttranslational modifications (PTMs) of the ATRA receptor RARα, and to assess its consequent impact on tumour progression in salivary adenoid cystic carcinoma (SACC).

**Methods:**

Transcriptome sequencing was performed on public datasets from the Gene Expression Omnibus (GEO) and on clinical tissue samples to identify overlapping differentially expressed genes. The expression pattern of PRAME across human pan‐cancer and its correlation with overall survival were analyzed using the TCGA database. Cellular transcriptome sequencing, cellular functional assays, co‑immunoprecipitation (Co‐IP), western blotting, protein stability assays, nude mouse xenograft tumour and lung metastasis models, and bioinformatic analyses were conducted to investigate the potential regulatory mechanism by which PRAME promotes metastasis in SACC through degradation of RARα and regulation of the EMT pathway.

**Results:**

PRAME was found to be highly expressed in 33 common human tumour types, with minimal expression in normal tissues, and its upregulation correlated with poor prognosis. In SACC clinical specimens and cell lines, PRAME expression was significantly elevated. Functionally, PRAME upregulated Twist1 expression and promoted epithelial–mesenchymal transition, thereby enhancing the invasive and metastatic capabilities of SACC cells. In vivo, PRAME silencing attenuated subcutaneous tumorigenesis and pulmonary metastasis. Mechanistically, PRAME bound to the Cullin2 ubiquitin ligase complex, destabilizing RARα protein and promoting epithelial–mesenchymal transition (EMT).

**Conclusion:**

Collectively, our findings reveal that PRAME drives SACC metastasis by accelerating RARα degradation through the Cullin2 ubiquitin–proteasome pathway while simultaneously inducing Twist1‐mediated EMT. These results position PRAME as a candidate biomarker and an actionable therapeutic target in SACC.

**Key points:**

- In salivary adenoid cystic carcinoma (SACC), PRAME drives tumour cell invasion and metastasis by upregulating Twist1 and inducing epithelial–mesenchymal transition.
- PRAME associates with the Cullin2 ubiquitin ligase to destabilize RARα, uncovering a posttranslational mechanism that may limit ATRA responsiveness in SACC.
- PRAME promotes subcutaneous tumour growth and pulmonary metastasis in vivo, consolidating its role as a key oncogenic driver in SACC.

## Graphical abstract

PRAME promotes SACC invasion and metastasis by inducing EMT via Twist1 upregulation and by destabilizing RARα through Cullin2‑mediated ubiquitination, a mechanism that may limit ATRA efficacy. In vivo, PRAME enhances subcutaneous growth and lung metastasis, highlighting its oncogenic role in SACC.

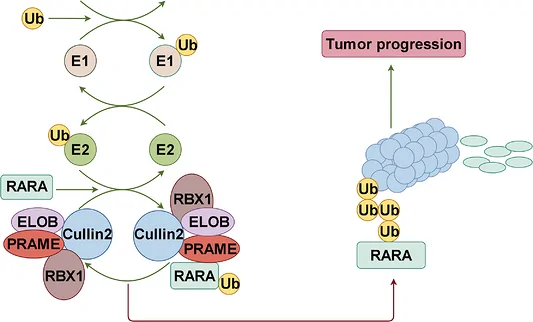

## 1 INTRODUCTION

Salivary adenoid cystic carcinoma (SACC) is a type of malignant tumour with specific clinical and pathological features, comprising around 10% of all salivary gland neoplasms and 3%–5% of head and neck carcinomas.^1^, ^2^ It mainly affects individuals aged 50‐60 years, and there is a slight female majority.^3^ SACC demonstrates slow growth but significant invasiveness, often showing perineural invasion and a strong tendency for distant metastasis,^4^, ^5^ especially to the lungs (40%–50% of cases).^6^, ^7^ While surgery combined with radiotherapy can effectively manage local disease, the long‐term prognosis remains unfavourable, with a 20‐year survival rate below 30%.^8^ This unfavourable result is mainly due to the difficult management of distant metastasis.^9^, ^10^ Recent comprehensive genomic profiling has further elucidated the complex mutational landscape of ACC, reinforcing the need for novel therapeutic targets.^11^ So, understanding the molecular mechanisms behind SACC progression and metastasis is super important for developing new therapeutic strategies and making patient survival better.

Cancer‑testis antigens (CTAs) comprise a family of proteins aberrantly overexpressed in diverse tumour types, whereas their expression in normal tissues is largely confined to immune‑privileged sites such as the testis.^12^, ^13^ These antigens play a dual role by influencing tumour cell proliferation, resistance to apoptosis, and metastatic ability, while also triggering specific immune responses, making them promising candidates for cancer immunotherapy.^14^, ^15^ The Cullin2 ubiquitin ligase complex is a crucial part of the ubiquitin–proteasome system, which selectively regulates the stability of oncoproteins and tumour suppressors by recognizing specific substrates through distinct adaptors like FEM1B and ZYG1B.^16^, ^17^ The modular architecture of this system further provides a critical molecular framework for developing targeted protein degradation technologies, including proteolysis‑targeting chimaeras (PROTACs).^18^, ^19^

Notably, approximately 80% of SACC cases have genomic alterations in MYB or MYBL1, which shows the importance of MYB‐like signalling pathways in SACC initiation and maintenance.^20^ All‐trans retinoic acid (ATRA) has been demonstrated to inhibit MYB activity.^21^ Its biological functions mainly rely on heterodimers, comprising retinoic acid receptors (RAR‐α, RAR‐β, RAR‐γ) and retinoid X receptors (RXR).^22^ Among these, RARα is a key player, a regulator of cellular differentiation and proliferation. Its dysregulation is closely linked to tumorigenesis and progression, and it is also the receptor subtype most intimately involved in ATRA signal transduction.^23^, ^24^, ^25^ However, the therapeutic efficacy of ATRA in solid tumours including SACC remains limited, and overcoming this resistance represents a major challenge in contemporary oncology research.^21^, ^26^

## 2 MATERIALS AND METHODS

### 2.1 Tissue collection and ethical statement

Clinical tissue specimens were obtained from surgically resected tumour tissues of SACC patients at Beijing Tongren Hospital, with involved sites including the nasal cavity, paranasal sinuses, parotid gland, and lung metastases, as well as from adjacent normal tissues at surgical margins. Patients with prior preoperative radiotherapy or chemotherapy were not included. Approval for the study was obtained from the Institutional Ethics Committee of Beijing Tongren Hospital, Capital Medical University (ethics approval no.: TREKY2020‐021).

### 2.2 Cell lines and cell culture

The SACC‐LM and SACC‐83 cell lines were sourced from Peking University School of Stomatology. Cell line authentication was performed using short tandem repeat (STR) profiling, and mycoplasma contamination was excluded. Cells were incubated at 37°C in a humidified atmosphere with 5% CO_2_. RPMI‑1640 (Gibco, USA) containing 10% fetal bovine serum (Gibco, Australia) and 1% penicillin/streptomycin (Gibco) was used as the culture medium.

### 2.3 siRNA transfection

Cells were seeded in six‐well plates. Lyophilized RNA oligonucleotides (GenePharma, Shanghai, China) were briefly centrifuged and reconstituted to a final concentration of 20 µM. For each well, 3.75 µL of the siRNA stock solution was used. Subsequently, 7.5 µL of siRNA‐mate plus transfection reagent was added to the siRNA/miRNA mixture. The resulting RNA/plus complex was gently mixed and added dropwise to the cells. The sequences of target siRNAs and negative controls are listed in Table S1.

### 2.4 Vector construction and cell transduction

The complete cDNA sequence of human PRAME was inserted into the Ubi‐MCS‐3FLAG‐CBh‐gcGFP‐IRES‐puromycin lentiviral vector (GV492, Genechem, China) for the purpose of gene overexpression. The hU6‐MCS‐CBh‐gcGFP‐IRES‐puromycin lentiviral vector (GV248, Genechem) was used to clone the shRNA that targets PRAME. According to a multiplicity of infection (MOI) of 10, the lentiviral stock solution and 40 µL HiTransG A/P infection enhancement solution (25×) were added to six‐well plates. After thorough mixing, cells were incubated for an additional 24 h, and the medium was replaced with fresh medium containing 2 µg/mL puromycin. The plasmid vector U6‐sgRNA‐EF1a‐spCas9‐FLAG‐CMV‐EGFP‐P2A‐puro (GV708, Genechem, China), which contains BsmBI sites, was used to clone the sgRNA targeting *RARA*. The resulting recombinant vector was confirmed by DNA sequencing. The resulting construct was introduced into *E. coli* DH5α, and the plasmid was purified with the Endofree Mega Kit (Qiagen, Hilden, Germany) to generate sgRNA‐*RARA*. Target and control sequences are provided in Table S1.

### 2.5 RNA extraction and quantitative real‐time PCR (qRT‐PCR)

The process involved isolating total RNA from cells with TRIzol Reagent (Thermo, USA) and then synthesizing cDNA using the RevertAid First Strand cDNA Synthesis Kit (Thermo) as directed by the manufacturer. Reverse transcription consisted of incubation at 42°C for 60 min, followed by 70°C for 5 min, with a final hold at 4°C. Real‐time PCR was carried out with SYBR Green Master Mix (TOYOBO, China), and GAPDH served as the reference. The 2^−^^ΔΔC^ *^t^* approach was applied to calculate relative expression. Primer sequences are detailed in Supporting Information.

### 2.6 Co‐immunoprecipitation and immunoblotting

Co‐Immunoprecipitation (Co‐IP) experiments were performed using the Pierce co‐immunoprecipitation kit (Thermo). Antibody‐coupled resin was incubated overnight at 4°C. Cells were lysed in pre‐chilled IP lysis buffer and incubated on ice for 5 min with periodic mixing. The lysate was centrifuged for 10 min to pellet cellular debris. The resin was incubated overnight at 4°C with bait and target proteins or control samples. All Co‐IP procedures were conducted at 4°C. To prepare for SDS‐PAGE, samples were mixed with a 5× loading buffer to reach a 1× concentration, heated at 100°C for 5 min, and then cooled to room temperature. Antibody information is provided in Table S2.

### 2.7 Transwell migration and invasion assays

Matrigel (Beyotime, China) was diluted 1:8. Cells at exponential growth were harvested, diluted to 2.5 × 10^5^/mL, and subsequently transferred to upper chambers, either pre‐coated with Matrigel or left uncoated. Medium with 15% FBS was introduced to the lower chambers, followed by a 24 h incubation of the plates. To fix the cells, 4% paraformaldehyde was applied, followed by staining with 0.1% crystal violet for 20 min. The top surface of the chambers had cells removed with cotton swabs.

### 2.8 Wound healing assay

Cells were seeded in six‐well plates at a density of 2.5 × 10^5^ cells per well. Upon reaching approximately 95% confluence, a linear scratch was made across the monolayer using a 200 µL pipette tip. After washing with PBS, cells were cultured in serum‐free RPMI‐1640 medium for 24 h. An inverted microscope was used to capture images at both 0 and 24 h.

### 2.9 Haematoxylin and eosin (H&E) staining

Tissue specimens were embedded in paraffin and sectioned. After deparaffinization and rehydration through xylene and graded ethanol, the sections were stained with haematoxylin for 3 min, differentiated, and blued. Sections were rinsed with tap water, stained with eosin for 15 s, dehydrated through a series of ethanol concentrations, cleared in xylene, and mounted with neutral balsam. An optical microscope was used to acquire images.

### 2.10 Phalloidin staining

CoraLite Plus 488‐labelled phalloidin (Proteintech, China) was reconstituted to a working concentration of 200T/mL. SACC cells in logarithmic growth phase were washed with PBS, then treated with 4% paraformaldehyde on ice for 15 min. The cells were permeabilized at room temperature for 5 min using PBS with 0.2% Triton X‐100. A fluorescently labelled phalloidin working solution was added and incubated for 20 min at room temperature. Stained cells were visualized using fluorescence microscopy.

### 2.11 Protein stability assay

For cycloheximide (CHX) chase assays, cells were treated with 100 µg/mL CHX (MedChemExpress, USA) in combination with 10 µmol/L DMSO or MG‐132 (MedChemExpress). RARα protein expression was evaluated by Western blotting in cells collected at the indicated time intervals.

### 2.12 Immunohistochemistry (IHC)

Paraffin sections were deparaffinized, rehydrated, and subjected to heat‐induced antigen retrieval. Endogenous peroxidase activity was blocked with 3% H_2_O_2_. Sections were blocked with 3% BSA at room temperature for 30 min and incubated with appropriately diluted primary antibodies overnight at 4°C. Following PBS washing, HRP‐linked secondary antibodies were added and incubated at room temperature for 50 min. A newly prepared DAB chromogen solution was added dropwise following PBS washing. The chromogenic reaction was monitored microscopically and terminated by rinsing with tap water when positive signals appeared brownish‐yellow. Nuclei were first counterstained with haematoxylin, and then differentiated and blued. Subsequent sections were then dehydrated, cleared, and mounted. Images were captured using an optical microscope.

### 2.13 Immunofluorescence (IF)

Cells cultured on 12‐well slides were washed with PBS and fixed with 4% paraformaldehyde for 15 min. Sections were probed with PRAME antibody at 4°C overnight, then labelled with green‐fluorescent secondary antibody. The same sections were subsequently incubated with RARα antibody overnight at 4°C, followed by red‐fluorescent secondary detection. PBST washes were performed thoroughly before and after each antibody incubation. Finally, an anti‐fade mounting medium with DAPI (Beyotime) was used to mount the slides.

### 2.14 Animal experiments

All animal procedures were undertaken in the SPF barrier animal facility at the Institute of Animal Sciences, Chinese Academy of Medical Sciences. Male BALB/cA nude mice (aged 4–6 weeks) were obtained from HFK Bioscience Limited Company (Beijing, China). For subcutaneous xenograft establishment, 3.75 × 10^6^ stably transduced SACC‐LM cells were resuspended in 150 µL of phosphate‑buffered saline (PBS) and Matrigel, and subcutaneously injected into the right axilla of nude mice. After 20 days, all tumour tissues were harvested for subsequent analyses. Tumour volume was calculated using the formula: Volume (mm^3^) = length × width^2^ × 0.5. Additionally, a total of 200 µL of cell suspension containing 2 × 10^5^ SACC‑LM cells at the logarithmic growth phase was injected into the tail vein of mice. On day 21 postinjection, D‑luciferin potassium salt solution (15 mg/mL) was prepared, and mice were subjected to in vivo bioluminescence imaging using an IVIS Lumina III imaging system (PerkinElmer, USA). Animal experiments were approved by the Ethics Committee of the Institute of Animal Sciences, Chinese Academy of Medical Sciences (GR25002) and conformed to the Declaration of Helsinki guidelines.

### 2.15 Bioinformatics analysis

Transcriptome datasets GSE57901 and GSE88804 were sourced from the GEO database, and the edgeR package in R (version 4.4.3) was utilized to identify differentially expressed genes (DEGs). Analysis of PRAME expression levels across human pan‐cancer types was based on the TCGA database.^27^ The ubiquitination database was used to predict the E3 ligases that act on RARα.^28^ The STRING database (https://cn.string‐db.org/) was employed for protein–protein interaction analysis, and GSEA software was employed to analyze transcriptome sequencing data from SACC cells.^29^ Molecular docking for protein interaction sites was performed using the HDOCK program, and the reliability of docking conformations was assessed by the docking score. The criterion for reliable docking was a score of <−200, with lower scores indicating higher reliability.

### 2.16 Statistical analysis

GraphPad Prism (version 8.0, GraphPad Software, USA) was used to conduct the statistical analysis. Comparisons between two groups for qRT‑PCR assays, subcutaneous xenograft tumour weights, and high‑throughput sequencing data were performed using unpaired t‑tests. Comparisons among multiple groups for wound‐healing assays, transwell assays, subcutaneous xenograft tumour growth curves, and western blotting were assessed by two‐way ANOVA. Correlation between the mRNA expression levels of *PRAME* and *RARA* expression in primary tumours and lung metastases was evaluated using Spearman's rank correlation. All experiments were performed with at least three independent replicates. Significance levels were denoted as *p < .05, **p < .01, ***p < .001, ****p < .0001; ns, not significant.

## 3 RESULTS

### 3.1 PRAME is highly expressed in SACC and pan‐cancer and correlates with poor prognosis

**FIGURE 1:**
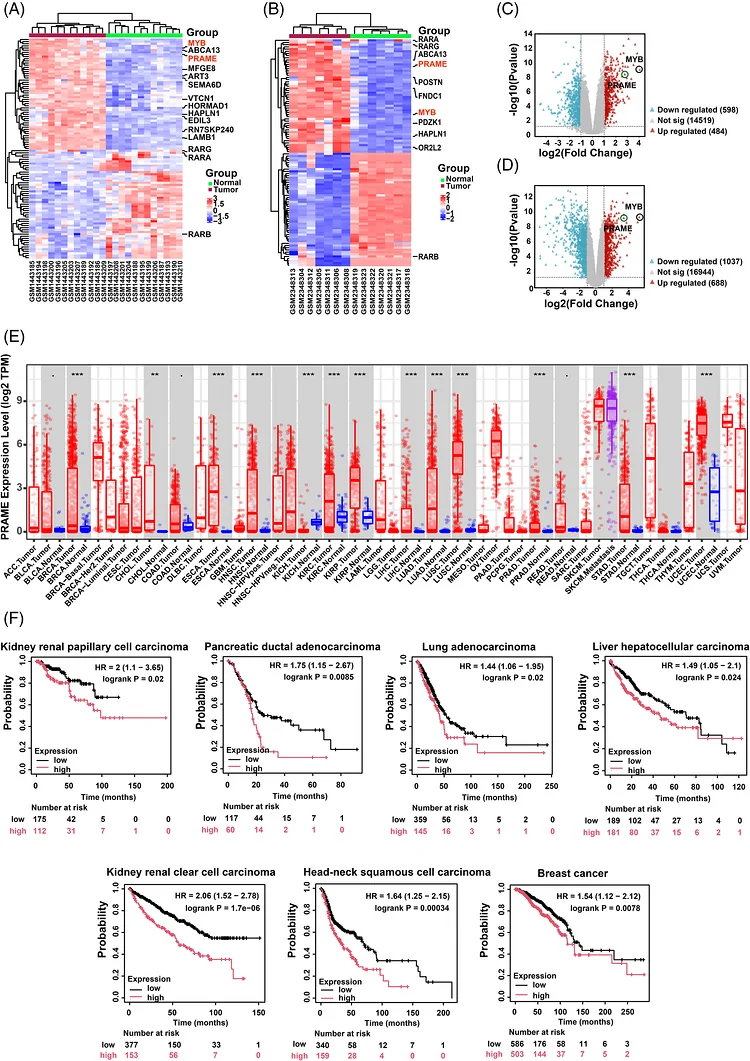
PRAME is significantly overexpressed across human pan‐cancer and correlates with poorer overall survival. (A, B) Heatmap analysis of expression profile microarrays from the GEO database. The X‑axis corresponds to sample identifiers, the Y‐axis to gene symbols. PRAME and MYB are highlighted in red. (C, D) Volcano plot analysis of expression profile microarrays from the GEO database. Green circles denote PRAME, black circles indicate MYB. (E) PRAME mRNA expression across 33 human tumour types based on TCGA data, presented as Log_2_ (TPM) values. Red, blue, and purple bars represent primary tumour, normal, and metastatic tumour tissues, respectively. (F) PRAME expression is a significant risk factor for overall survival in pan‐cancer based on TCGA analysis. Red and black curves represent high and low PRAME expression groups, respectively.

**FIGURE 2:**
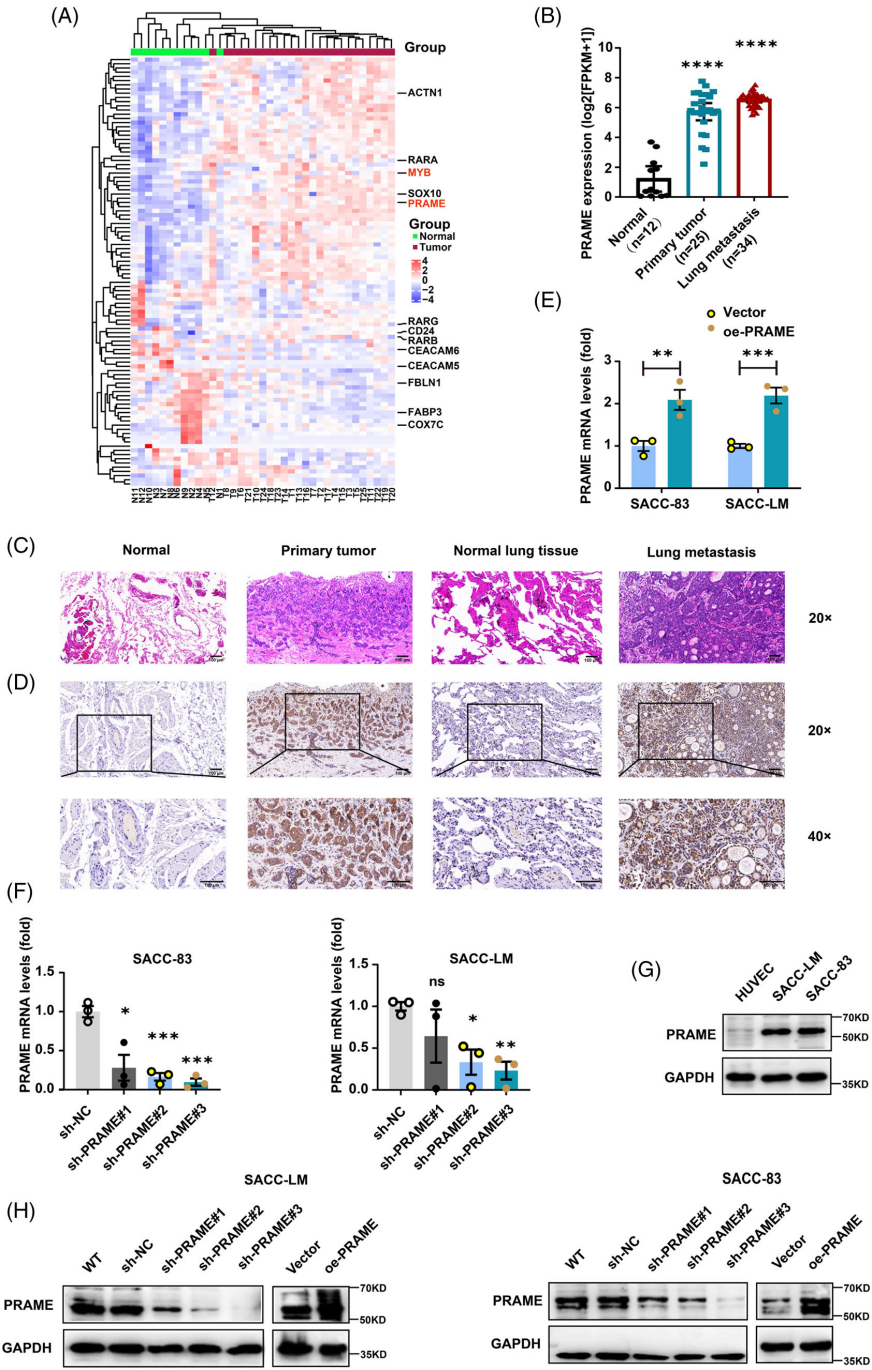
PRAME expression is significantly upregulated in SACC clinical specimens and cell lines. (A) Heatmap comparing normal (*n* =  12) and tumour (*n* =  25) groups based on high‐throughput sequencing of clinical specimens. (B) PRAME mRNA levels in clinical specimens across different tissue types determined by high‐throughput sequencing, expressed as Log_2_(FPKM+1). Data are shown as mean  ±  SD. Statistical analysis is performed using unpaired *t*‑test (*p < .05, **p < .01, ***p < .001, ****p < .0001). (C) H&E staining of serial paraffin sections from clinical samples of different tissue types. (D) PRAME expression in different clinical tissues detected by IHC staining on serial paraffin sections. Scale bars: 100 µm (20× and 40× magnifications). (E, F) qRT‑PCR analysis of PRAME mRNA levels in SACC cells with stable lentiviral transduction, confirming overexpression and knockdown efficiency. (G) Western blot analysis of PRAME protein expression in normal and SACC cells. (H) Verification of PRAME knockdown and overexpression at the protein level in SACC cells by Western blotting. WT, wild‑type.

To identify key factors involved in SACC pathogenesis, we analyzed high‐throughput datasets from the GEO database. In the GSE59701 dataset (12 tumour and 12 normal samples), 484 significantly upregulated and 598 downregulated genes were identified. In the GSE88804 dataset (7 tumour and 7 normal samples), 688 upregulated and 1037 downregulated genes were identified. Corresponding heatmap and volcano plot visualizations are shown in Figure 1A–D. Pan‐cancer analysis revealed that PRAME is broadly overexpressed across 33 major tumour types, with negligible expression in normal tissues (Figure 1E). Kaplan–Meier analysis of TCGA data further confirmed that high expression of PRAME is significantly connected to worse overall survival outcomes (Figure 1F). In our clinical cohort (25 primary tumours, 34 lung metastases, and 12 normal tissues), transcriptomic sequencing showed a stepwise increase in PRAME expression from normal tissues to primary ACC tumours and lung metastases, with levels in both tumour types being significantly higher than in normal tissues (Figure 2A,B). These results, together with public database analyses, indicated that PRAME expression abundance in SACC closely paralleled that of the signature gene MYB. IHC staining of clinical samples revealed significantly elevated levels of PRAME protein in primary tumours and lung metastases compared with adjacent normal tissues (Figure 2C,D). These findings collectively indicate that PRAME is overexpressed specifically in tumours, and this overexpression is inversely associated with overall survival.

### 3.2 PRAME promotes migration and invasion of SACC cells

**FIGURE 3:**
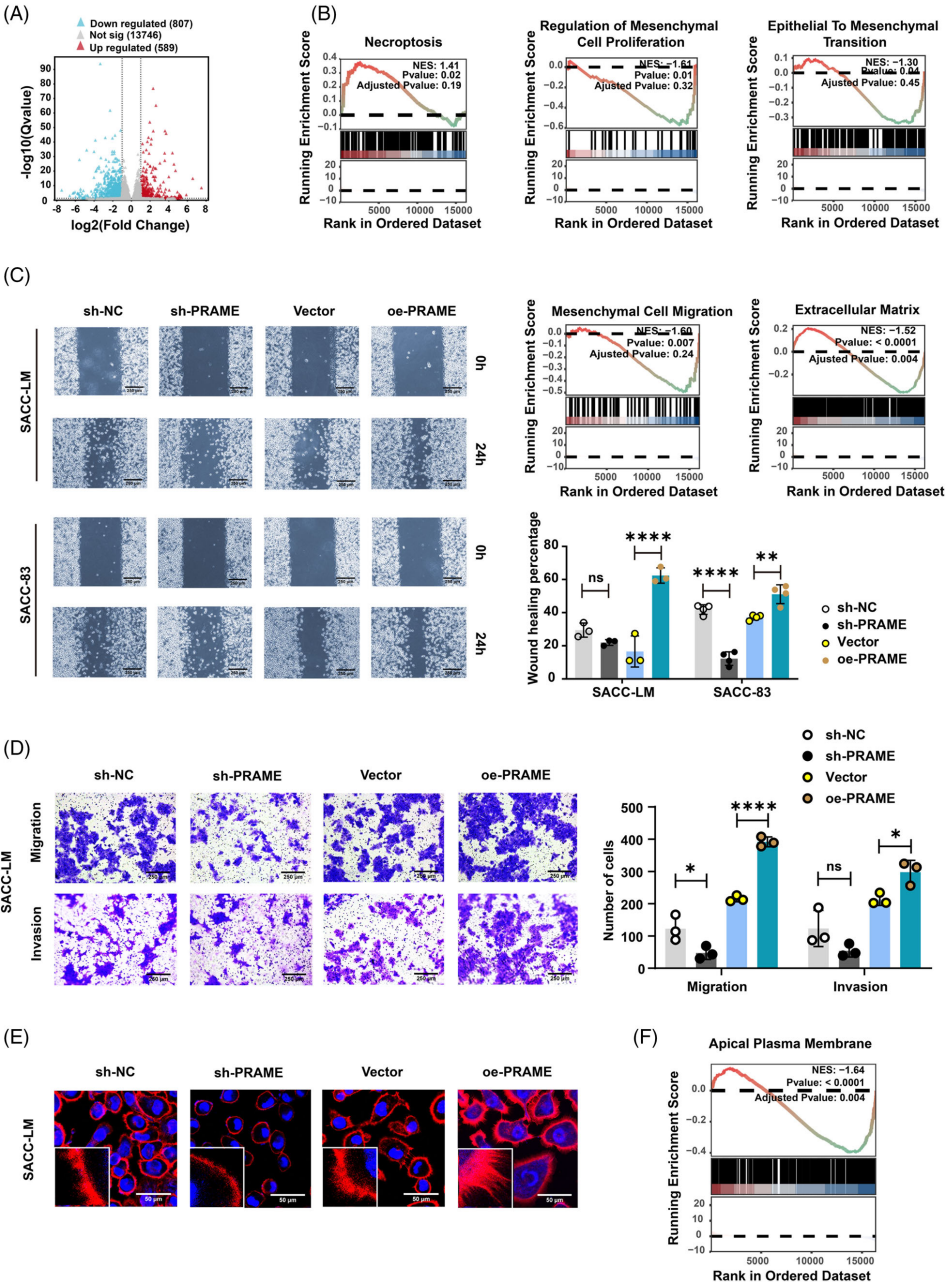
PRAME promotes migration and invasion in SACC cells. (A) Volcano plots based on high‐throughput sequencing of SACC cells stably transduced with sh‐NC (*n* =  4) and sh‐PRAME (*n* =  4). (B) Gene set enrichment analysis (GSEA) of high‐throughput data from SACC cells identifies PRAME‐associated alterations in extracellular matrix status and apoptosis function. (C) Wound healing assay assessing the effect of PRAME on SACC cell motility, with quantitative analysis (bottom right). Data are shown as mean  ±  SD. Statistical analysis is performed using two‑way ANOVA. (D) Transwell assay evaluating the effects of PRAME on migration and invasion of SACC cells. Scale bar: 250 µm. Statistical analysis is performed using two‑way ANOVA (*p < .05, **p < .01, ***p < .001, ****p < .0001; ns, not significant). (E) Phalloidin staining examining the influence of PRAME on filopodia formation in SACC cells. Scale bar: 50 µm. (F) High‐throughput sequencing and GSEA of sh‐NC and sh‐PRAME SACC cells reveal PRAME‐associated effects on the plasma membrane.

To investigate the functional role of PRAME, stable SACC‐83 and SACC‐LM cell lines with PRAME overexpression or knockdown were generated. Three independent shRNA sequences targeting PRAME were designated sh‐PRAME#1, #2, and #3. Knockdown and overexpression efficiencies were validated at both mRNA and protein levels (Figure 2E,F,H). PRAME expression in SACC cells was substantially higher than that in HUVECs (Figure 2G). Additionally, transcriptome sequencing of sh‐NC and sh‐PRAME SACC cells (*n* = 4 each) identified 589 upregulated and 807 downregulated genes as candidate downstream targets (Figure 3A). Enrichment analysis revealed a dual role for PRAME in suppressing necroptosis and driving mesenchymal cell behaviours, including proliferation, migration, and EMT (Figure 3B). Wound healing and Transwell assays confirmed that PRAME augments the ability of SACC cells to migrate and invade (Figure 3C,D). Furthermore, sequencing analysis indicated that PRAME drives alterations in the apical plasma membrane (Figure 3F), and phalloidin staining demonstrated that PRAME promotes the formation of filopodia (Figure 3E). Together, these data show that PRAME contributes to the malignant phenotypes in SACC cells.

### 3.3 PRAME interacted with RARα and downregulated its protein expression

**FIGURE 4:**
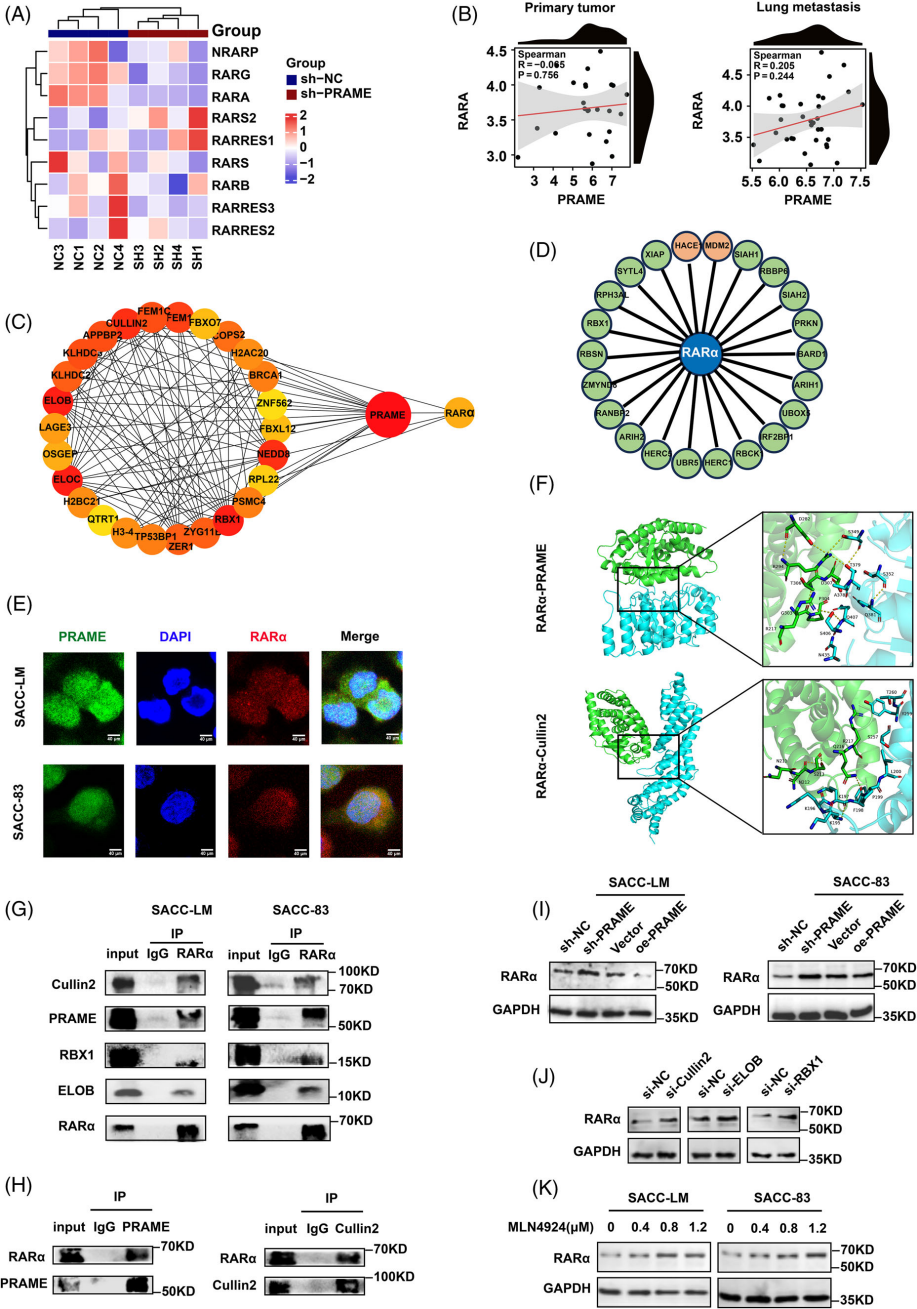
Figure 4 PRAME interacted with RARα and downregulated its protein level. (A) High‐throughput sequencing of SACC cells with heatmap visualization; colour intensity represents Log_2_FC values. (B) RNA‐level correlation between PRAME and RARα in clinical samples analyzed by high‐throughput sequencing. Statistical analysis is performed using the Spearman test. (C) PPI analysis based on protein databases; colour intensity indicates the number of interacting proteins. (D) Ubiquitin ligases targeting RARα identified from protein databases. Orange and green indicate previously reported and predicted interactions, respectively. (E) Immunofluorescence (IF) analysis of PRAME and RARα subcellular localization in SACC cells. PRAME is shown in green, RARα in red. Scale bar: 40 µm. (F) Molecular docking to predict the interaction sites and calculate the docking scores for RARα‐PRAME and RARα‐Cullin2. (G) Endogenous co‑immunoprecipitation with RARα antibody and Western blotting in wild‐type SACC cells. (H) Endogenous co‑immunoprecipitation with PRAME or Cullin2 antibody followed by Western blotting for RARα in SACC cells. (I) Western blotting validation of PRAME and RARα expression correlation in stably transduced SACC cell lines. (J) RARα protein levels in SACC cells following transfection with siRNAs targeting Cullin2, ELOB, and RBX1. (K) RARα protein expression under different concentrations of MLN4924, with GAPDH as loading control.

ATRA has been reported to inhibit MYB expression, but it is not an effective monotherapy for SACC.^10^, ^30^ Given that RARα is the main receptor involved in ATRA signalling, we analyzed the regulatory linkages between PRAME and RARα. Transcriptomic sequencing of SACC cells revealed no significant correlation between PRAME and RARα expression at the mRNA level (Figure 4A), and this finding was validated by sequencing data from primary and metastatic SACC tissue samples (Figure 4B). But STRING database analysis predicted that protein‐level interactions exist among RARα, PRAME, and NEDD8, and also associations exist between PRAME and Cullin2, ELOB, and RBX1 (Figure 4C). Ubiquitination database mining further identified RBX1 as a candidate E3 ligase for RARα (Figure 4D). Immunofluorescence showed co‐localization of PRAME and RARα in SACC cells (Figure 4E). Molecular docking indicated that, between the two systems (RARα‐PRAME and RARα‐Cullin2), model 1 yielded the highest absolute docking scores, with values of –236.53 and –237.26, respectively, indicating reliable interaction stability (Figure 4F). Furthermore, endogenous co‑immunoprecipitation combined with western blotting confirmed that RARα interacted with Cullin2, PRAME, RBX1, and ELOB (Figure 4G,H). Western blotting confirmed an inverse correlation between PRAME and RARα protein levels (Figure 4I). Additionally, transient siRNA‑mediated knockdown of Cullin2, ELOB, and RBX1 individually significantly increased RARα protein expression in cells, suggesting a potential negative regulatory relationship between these proteins and RARα (Figure 4J). Treatment with MLN4924, an inhibitor of NEDD8‐activating enzyme that blocks Cullin‐RING ligase activity, led to RARα accumulation (Figure 4K). Collectively, these results indicated that PRAME may regulate post‑translational modifications of RARα.

### 3.4 PRAME mediates ubiquitination and degradation of RARα via the Cullin2‑proteasome system

**FIGURE 5:**
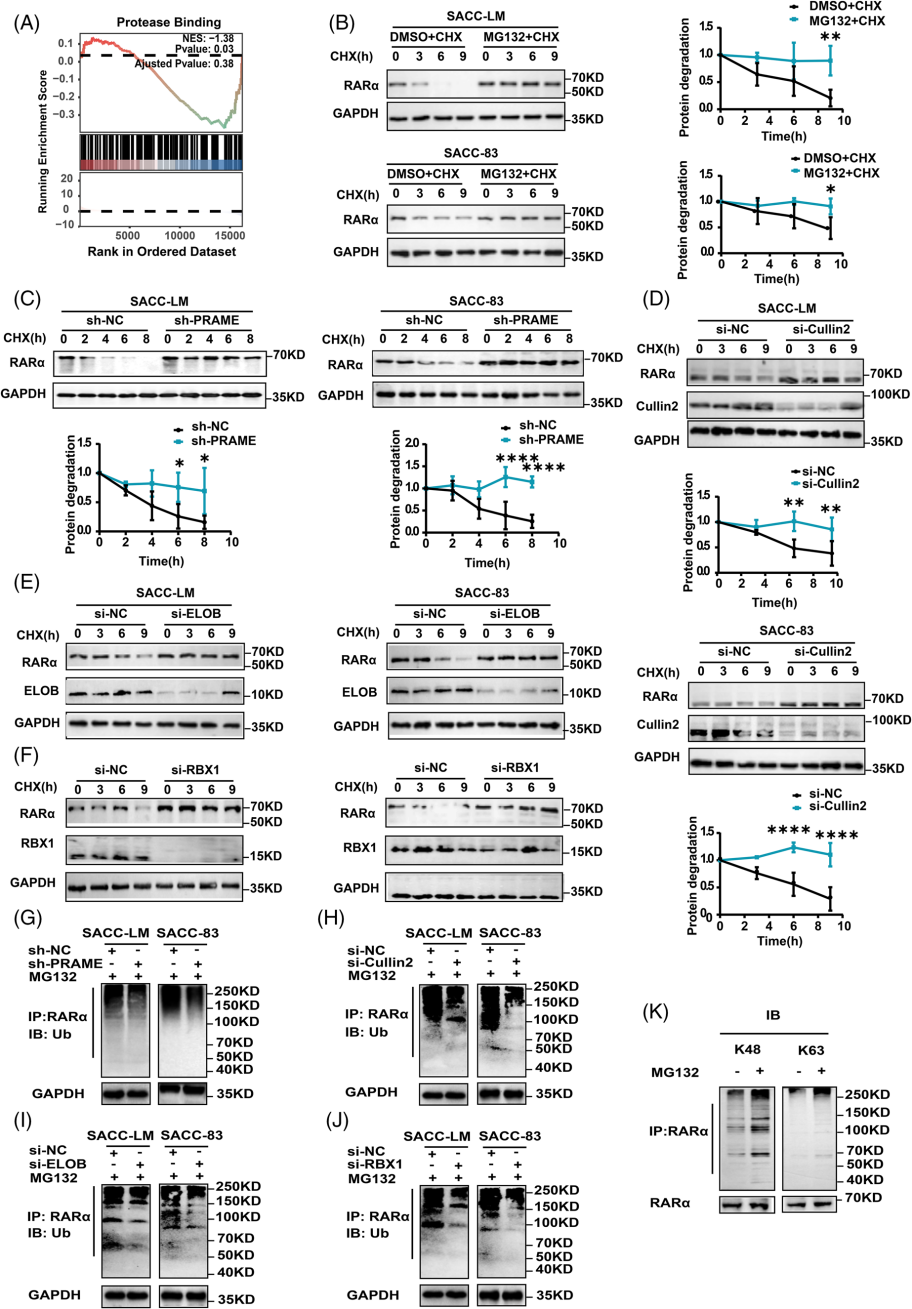
Figure 5 PRAME promotes ubiquitination and degradation of RARα via the Cullin2‐proteasome system. (A) High‐throughput sequencing and GSEA of sh‐NC and sh‐PRAME SACC cells validate the effect of PRAME on ubiquitin‐proteasome degradation. (B) RARα protein stability under 100 µg/mL CHX with 10 µmol/L DMSO or MG‐132. The right panel represents the quantitative statistical analysis. (C) PRAME and RARα expression in sh‐NC and sh‐PRAME SACC cells treated with 100 µg/mL cycloheximide (CHX) at the indicated time points. Quantification is shown in the lower panel. (D–F) SACC cells transiently transfected with the indicated siRNAs were pretreated with MG‐132 for 2 h, lysed, and subjected to immunoprecipitation with RARα antibody, followed by Western blotting for ubiquitin. (G) SACC cells stably transfected with sh‐NC or sh‐PRAME were pretreated with MG‐132 for 2 h, followed by immunoprecipitation with anti‐RARα antibody and detection of RARα ubiquitination using anti‐ubiquitin antibody. (H–J) SACC cells transiently transfected with siRNAs against Cullin2, ELOB, and RBX1, pretreated with MG‐132 (2 h), followed by RARα immunoprecipitation and ubiquitination detection with anti‐ubiquitin antibody. (K) Wild‐type SACC cells, with or without MG‐132 pretreatment (2 h), subjected to RARα immunoprecipitation and ubiquitination detection using K48‐ and K63‐specific antibodies.

E3 ligases act as the primary arbiters of substrate specificity in ubiquitin‐proteasome degradation.^31^ Among them, Cullin‐RING E3 ligases (CRLs) account for approximately 20% of cellular protein degradation.^32^, ^33^ GSEA of transcriptome sequencing data from SACC cells with sh‑NC or sh‑PRAME indicated that PRAME enhanced proteasome binding and consequently facilitated ubiquitination (Figure 5A). Wild‐type SACC cells treated with cycloheximide (CHX) exhibited decreased RARα protein stability over time, whereas the half‐life of RARα was prolonged upon MG132 treatment (Figure 5B). RARα protein stability was increased in the PRAME‐knockdown group. Additionally, Cullin2, ELOB, and RBX1 were individually knocked down by transient siRNA transfection. CHX chase assays further demonstrated that knockdown of these genes prolonged the half‐life of RARα and thereby improved its stability (Figure 5D–F). Finally, PRAME, Cullin2, ELOB, and RBX1 were individually knocked down by transfection in SACC cell lines. Co‐IP assays showed that silencing these complex components inhibited polyubiquitination of RARα (Figure 5G–J). Assessment with K48‐ and K63‐specific ubiquitin antibodies revealed that MG132 suppressed ubiquitin‐mediated degradation of RARα, with K48‐linked ubiquitination being the predominant type. Collectively, the PRAME‐RBX1‐Cullin2‐ELOB complex associates with RARα and mediates its ubiquitination and subsequent proteasomal degradation.

### 3.5 PRAME promotes tumour formation and lung metastasis in vivo by regulating RARα and the EMT pathway

**FIGURE 6:**
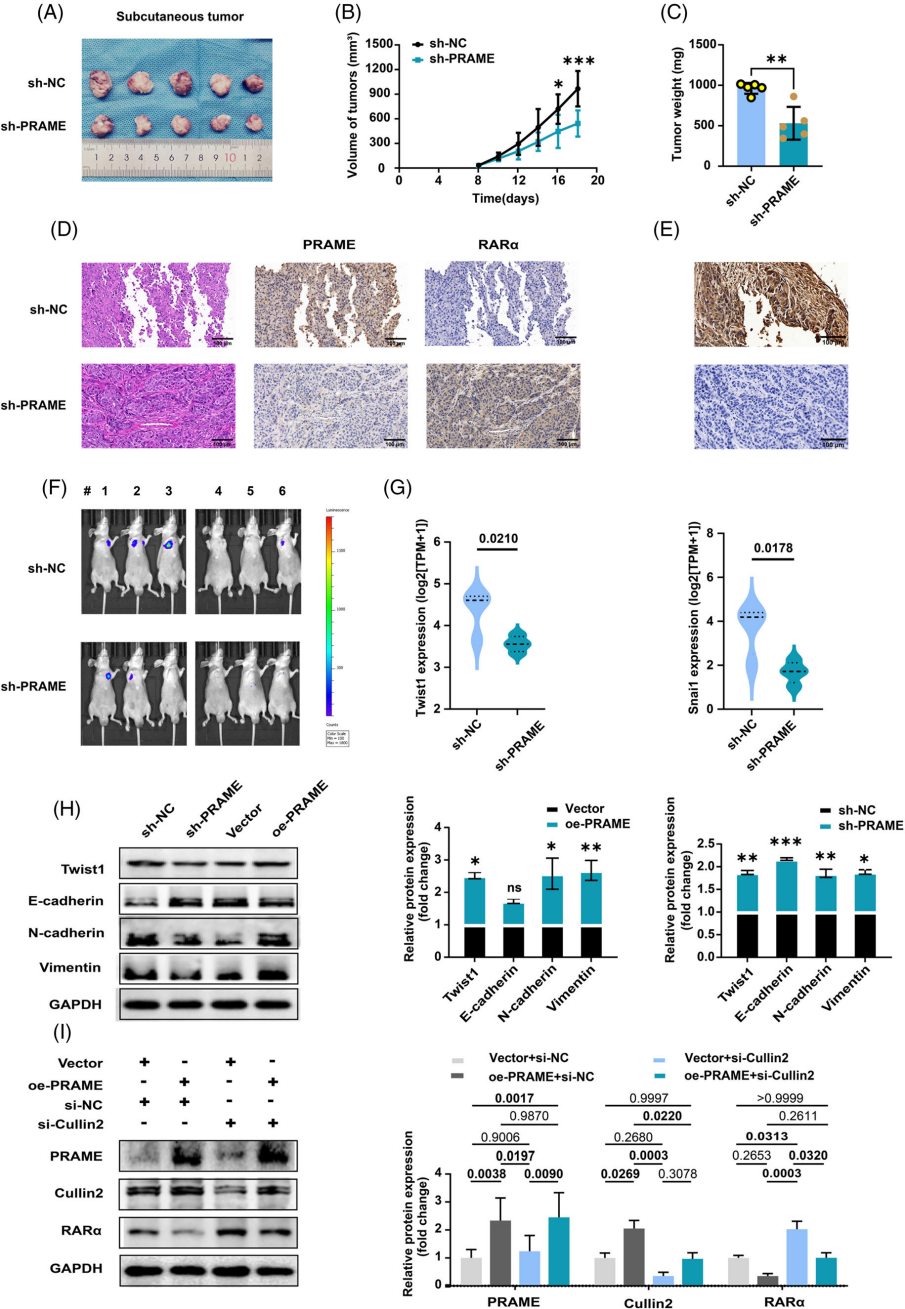
Figure 6 PRAME promotes subcutaneous tumour formation and lung metastasis in mice by regulating RARα and the EMT pathway. (A) Gross images of subcutaneous xenograft tumours in nude mice (*n* = 5 per group). (B) Growth curves of the tumours. Data are presented as mean ± SD. (C) Tumour weights (mean ± SD). *p < .05, **p < .01, ***p < .001; ns, not significant. (D) H&E and IHC staining of serial paraffin sections; scale bar = 100 µm. (E) Positive and negative controls for IHC staining on paraffin sections. (F) Nude mice were imaged in vivo at 21 days after tail‐vein injection of SACC‐LM cells. Colour scale: min = 100, max = 1800. (G) RNA‐seq analysis of sh‐NC and sh‐PRAME SACC cells revealed the mRNA expression levels of EMT transcription factors, presented as Log_2_(TPM+1). An unpaired *t*‐test was used for statistical comparisons. (H) Twist1 upregulation and EMT promotion by PRAME were confirmed at the protein level in stably transfected SACC cells. Quantitative data are shown on the right. All raw data were initially normalized to the average of the control group (vector or sh‐NC), which was set to 1.0, to represent relative fold changes. (I) (Left) Western blotting validation of PRAME‐mediated downregulation of RARα protein expression through Cullin2. (Right) Corresponding quantification with *p*‐values.

To assess the role of PRAME in tumorigenesis in vivo, SACC cells stably expressing sh‑NC or sh‑PRAME were implanted subcutaneously into nude mice. PRAME knockdown markedly inhibited tumour growth, as reflected by significant reductions in both tumour volume and weight compared with the control group (Figure 6A–C). IHC staining of paraffin‐embedded subcutaneous xenograft sections revealed that RARα protein expression was upregulated in PRAME‐knockdown tumours (Figure 6D). Subsequently, in vivo validation in mice injected via the tail vein with sh‐NC or sh‐PRAME SACC cells demonstrated that PRAME knockdown reduced the incidence of lung metastasis (Figure 6F). Furthermore, RNA‐seq analysis of sh‐NC and sh‐PRAME SACC cells showed that knockdown of PRAME significantly decreased the mRNA expression levels of Twist1 and Snail1, key transcription factors governing the EMT program (Figure 6G). By Western blotting, it was observed that Twist1, N‐cadherin, and Vimentin were downregulated, while E‐cadherin was upregulated upon PRAME knockdown. The EMT phenotype was restored by PRAME overexpression (Figure 6H). In summary, these results imply a potential mechanism through which PRAME regulates RARα to promote tumorigenesis and EMT, ultimately facilitating lung metastasis in vivo.

### 3.6 PRAME promotes invasion and migration of SACC cells via Cullin2 and RARα

**FIGURE 7:**
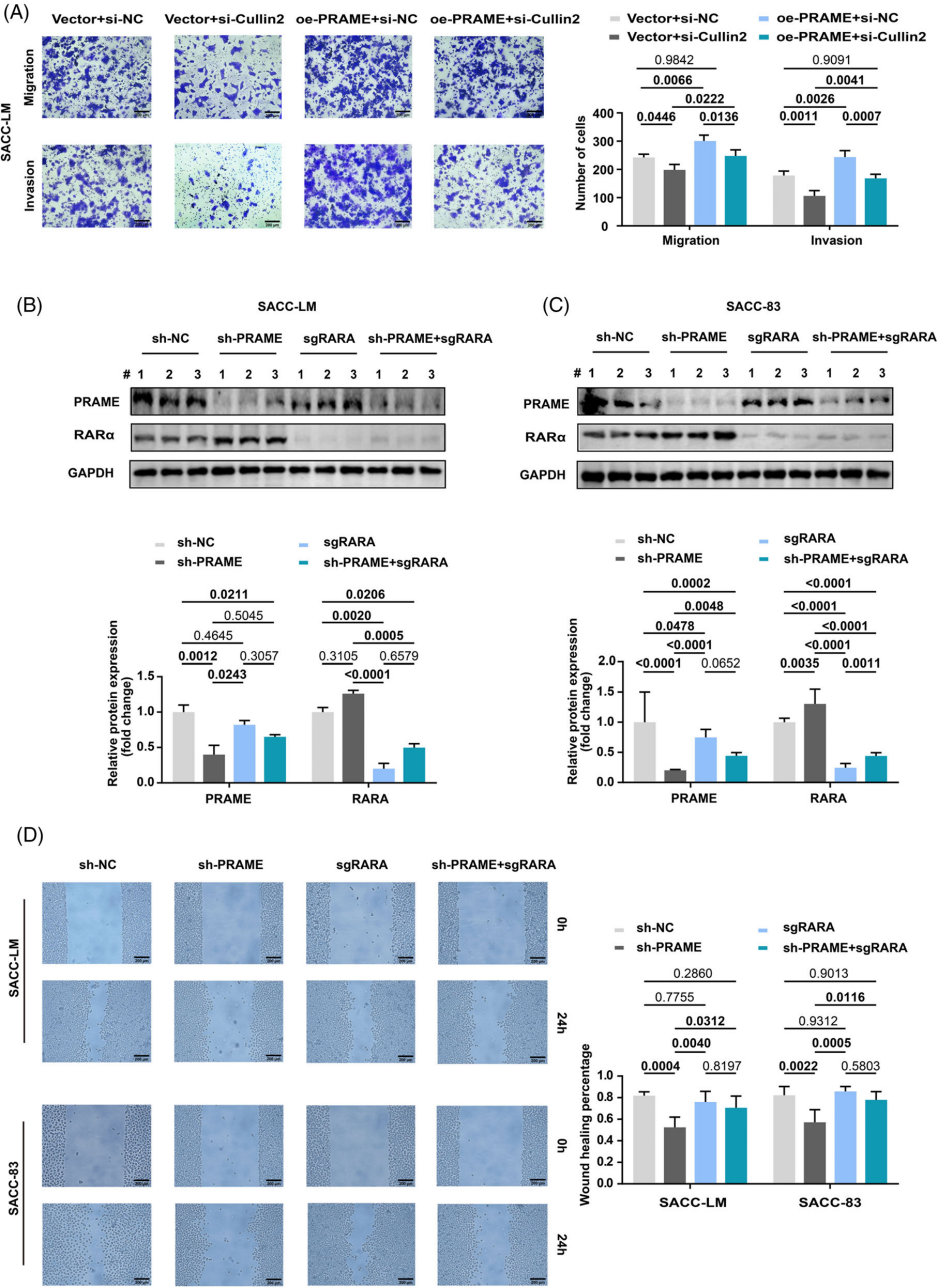
PRAME promotes SACC cell invasion and migration via Cullin2‐mediated downregulation of RARα. (A) Transwell assays assessing the invasion and migration of SACC‐LM cells mediated by PRAME through Cullin2. Quantitative data (mean ± SD) are presented in the right panel. (B, C) The efficiency of RARα knockout was confirmed at the protein level in SACC cell lines. Quantification is displayed in the lower panel, and the numbers in the figures denote *p*‐values. All raw data were initially normalized to the average of the control group (sh‐NC), which was set to 1.0, to represent relative fold changes. (D) Wound healing assay was performed to examine PRAME‐mediated SACC cell migration via RARα, with a scale bar of 200 µm. Quantitative results are presented in the right panel. Data are expressed as individual values with mean ± SD (*n* = 3 independent biological replicates).

In SACC cell lines stably transfected with empty vector or oe‐PRAME, we transiently knocked down Cullin2 using siRNA. Western blotting demonstrated that RARα protein expression was downregulated in the PRAME‐overexpressing group, and this reduction was restored upon Cullin2 knockdown (Figure 6I). Next, Transwell assays demonstrated that PRAME overexpression increased the invasion and migration abilities of SACC cells, whereas these phenotypes were rescued upon Cullin2 knockdown, with statistical significance (Figure 7A). Further, *RARA* was knocked out by plasmid transfection in sh‐PRAME SACC cells, and the knockout efficiency was confirmed at the protein level (Figure 7B,C). Wound healing assays demonstrated that PRAME knockdown decreased cell migration, whereas *RARA* knockout rescued this migratory phenotype (Figure 7D). Overall, these findings indicate that PRAME promotes the migratory capacity of SACC cells through Cullin2 and RARα.

## 4 DISCUSSION

Currently, the standard initial treatment for SACC is complete surgical resection, and then postoperative radiotherapy is performed.^3^, ^8^, ^34^ However, there are very few systemic therapeutic options for recurrent or metastatic disease.^10^ Clinical trials targeting NOTCH and c‐KIT pathways and immune checkpoint inhibitors generally result in low response rates.^35^, ^36^ For instance, recent trials of dual checkpoint inhibition have shown only modest disease control in recurrent/metastatic ACC.^37^ This is mainly because SACC have low levels of mutations in the tumour, reduced PD‐L1 expression, and a highly immunosuppressive microenvironment.^38^, ^39^ Hence, further dissection of the metastatic mechanisms in SACC and the pursuit of novel therapeutic targets remain of high clinical relevance.

PRAME, a prototypical cancer‐testis antigen (CTA), is upregulated in a lot of malignancies, like lung cancer, breast cancer, and melanoma.^40^, ^41^ Its expression level is linked to tumour aggressiveness and poor prognosis, and it has been demonstrated to facilitate immune evasion and distant metastases via several mechanisms.^42^ PRAME serves as a diagnostic marker in distinguishing in situ melanoma from dysplastic nevi.^43^ Therapeutically, PRAME‑based vaccines have been developed to elicit host antitumor immunity. In acute myeloid leukaemia (AML), dendritic cell (DC)‑based PRAME vaccines have demonstrated favourable safety profiles and immunogenicity.^44^ Adoptive cell therapy approaches have also been explored, in which autologous T cells are engineered to recognize and eliminate PRAME‑expressing tumour cells with high specificity. Among these, IMA203—a representative PRAME‑targeting TCR‑T therapy—has shown promising activity in melanoma, ovarian cancer, and synovial sarcoma.^45^ In this study, PRAME mRNA and protein expression were confirmed to be significantly upregulated in primary SACC tumours and lung metastases compared with normal tissues, using data from public databases and our clinical cohort. Moreover, PRAME expression was also markedly higher in SACC‐LM and SACC‐83 cell lines than in normal cell lines. Together, these observations suggest that PRAME may serve as an oncogene in SACC. The regulation of PRAME itself is complex, involving factors such as the long non‐coding RNA PRAME‐AS, which adds another layer of control to its oncogenic functions.^46^ Upon cellular uptake, all‐trans retinoic acid (ATRA) mainly binds to its nuclear receptor RARα and translocates to the nucleus to perform transcriptional functions.^47^ Although preclinical studies suggest that ATRA might suppress SACC progression by blocking the MYB signalling pathway,^48^ clinical trial data show that ATRA monotherapy only yields limited response rates in recurrent or metastatic SACC, with no significant prolongation of median progression‐free survival.^30^ Therefore, the potential mechanisms underlying the interplay between PRAME and ATRA receptors in SACC warrant further investigation. Ubiquitination represents a reversible post‐translational modification that dynamically governs the stability, activity, and localization of substrate proteins.^49^ This regulation is achieved through the concerted efforts of E3 ubiquitin ligases and deubiquitinating enzymes (DUBs).^31^ Disruptions in this regulatory system are commonly noted during the onset and progression of tumorigenesis.^50^, ^51^ For example, overexpression of the E3 ligase MDM2 causes the degradation of tumour suppressors like p53 to accelerate, thus promoting tumour growth.^52^ The Cullin2 ubiquitin ligase complex, a core component of the Cullin‐RING E3 ligase family, is widely involved in cell cycle control, hypoxia response, signal transduction, and tumour development through targeted degradation of key substrates.^16^, ^32^ In this study, *PRAME* and *RARA* showed no significant correlation at the mRNA level. However, through PPI analysis, molecular docking, and cycloheximide chase assays, we demonstrated for the first time that PRAME negatively regulates the stability of the retinoic acid receptor RARα via the Cullin2 ubiquitin‐proteasome system. This finding suggests a potential mechanism underlying the limited efficacy of ATRA in SACC. It is well established that EMT plays a critical role in tumorigenesis and progression, with Twist and Snail considered as pivotal transcription factors.^53^ Twist has been shown to promote migration, invasion, and metastasis, as well as to confer stem‐like traits and therapy resistance.^54^ In our study, PRAME was confirmed to promote EMT by upregulating Twist and Snail, which further supports its oncogenic role in SACC.

The cancer‐testis antigen is highly and specifically expressed in various tumours, while in normal tissues it is found only in reproductive tissues such as testes.^12^, ^13^ Consequently, it is considered a highly promising tumour‐specific immunotherapeutic target and has garnered widespread attention in fields such as T cell receptor therapy and CAR‐T.^14^, ^15^ This is exemplified by the recent development of T cell engagers specifically targeting PRAME peptide‐MHC complexes for solid tumour therapy.^55^ This study has several limitations. Given the indolent behaviour of SACC, which is highly prone to lung metastasis but progresses slowly, future studies with larger clinical cohorts and longer follow‐up are required to determine whether PRAME expression correlates with overall survival and progression‐free survival. Second, no specific PRAME‐targeted inhibitors are currently available; corresponding pharmacodynamic studies could not be performed in vivo; further investigations are warranted to evaluate the therapeutic applicability of targeting PRAME in physiological settings.

In conclusion, this study reveals a PRAME–Cullin2–RARα axis that drives EMT and metastasis in SACC, while also offering a novel posttranslational rationale for ATRA resistance.

## AUTHOR CONTRIBUTIONS

Xiaohong Chen, Lu Kong, and Xudong Wang conceived and designed the study. Xudong Wang performed the experiments under the supervision of Lu Kong and Ran Gao. Xudong Wang drafted the manuscript. Xiaohong Chen, Lu Kong, and Ran Gao supervised the revision of the manuscript. Xiaohong Chen, Lu Kong, and Ran Gao also contributed to project administration and experimental guidance. Tingyao Ma, Yixin Jing, Tianyi Li, Xi Zhao, and Guohan Zhao assisted in the collection of clinical specimens and conducting data analysis. All authors reviewed and approved the final manuscript.

## ETHICS STATEMENT

The study was approved by the Ethics Committee of Beijing Tongren Hospital, Capital Medical University (approval no. TREKY2020‐021). Animal experiments were conducted in accordance with the Declaration of Helsinki and approved by the Ethics Committee of the Institute of Animal Sciences, Chinese Academy of Medical Sciences (approval no. GR25002).

## CONFLICT OF INTEREST STATEMENT

The authors declare no conflict of interest.

## Supporting information



Supplementary Information

Supplementary Information

## Data Availability

The datasets generated during and/or analyzed during the current study are available from the corresponding author on reasonable request.
