# Supplementary material for: PRAME promotes invasion and metastasis of salivary adenoid cystic carcinoma by regulating RARα stability via the cullin2 ubiquitin‐proteasome system
Source: Clin Transl Med. 2026 Aug 27;16(9):e70787. doi: 10.1002/ctm2.70787 (PMC13521806; doi:10.1002/ctm2.70787)
Supplement: Supplementary file 1 [file CTM2-16-e70787-s001.docx]

**Table S1. Primers used in the experiments.**

| **Gene name** | **Primer sequence** | | **Application** |
| --- | --- | --- | --- |
| PRAME | F | 5′-CAGGACTTCTGGACTGTATGGT-3′ | qRT-PCR |
|  | R | 5′-CTACGAGCACCTCTACTGGAA-3′ |  |
| GAPDH | F | 5′-GGAGTCAACGGATTTGGT-3′ | qRT-PCR |
|  | R | 5′-GTGATGGGATTTCCATTGAT-3′ |  |
| si-NC | sense | 5′-UUCUCCGAACGUGUCACGUTT-3′ | si-RNA |
|  | anti-sense | 5′-ACGUGACACGUUCGGAGAATT-3′ |  |
| si-Cullin2 | sense | 5′-GCACAAUGCCCUUAUUCAATT-3′ | si-RNA |
|  | anti-sense | 5′-UUGAAUAAGGGCAUUGUGCTT-3′ |  |
| si-ELOB | sense | 5′-UGAACAAGCCGUGCAGUGATT-3′ | si-RNA |
|  | anti-sense | 5′-UCACUGCACGGCUUGUUCATT-3′ |  |
| si-RBX1 | sense | 5′-GACUUUCCCUGCUGUUACCUAA-3′ | si-RNA |
|  | anti-sense | 5′-UUAGGUAACAGCAGGGAAAGUC-3′ |  |
| sh-NC |  | 5′-TTCTCCGAACGTGTCACGT-3′ | sh-RNA |
| sh-PRAME#1 |  | 5′-GCCAGATGATTAATCTGCGTA-3′ | sh-RNA |
| sh-PRAME#2 |  | 5′-GCTGGACTCTATTGAAGATTT-3′ | sh-RNA |
| sh-PRAME#3 |  | 5′-CCTGTGATGAATTGTTCTCCT-3′ | sh-RNA |
| sg-NC  sg-RARA#1  sg-RARA#2  sg-RARA#3 |  | 5′-CGCTTCCGCGGCCCGTTCAA-3′  5′-ACACGCTGACGCCGGAGGTG-3′  5′-GAAGGTGCGCAAAGCGCACC-3′  5′-CGTGTAGCTCTCAGAGCACT-3′ | sg-RNA  sg-RNA  sg-RNA  sg-RNA |

Abbreviations: F: Forward; R: Reverse; qRT-PCR: quantitative real-time PCR; si-RNA: Small interfering-RNA; sh-RNA: Short hairpin-RNA; sg-RNA: Small guide-RNA.
