# Supplementary material for: PRAME promotes invasion and metastasis of salivary adenoid cystic carcinoma by regulating RARα stability via the cullin2 ubiquitin‐proteasome system
Source: Clin Transl Med. 2026 Aug 27;16(9):e70787. doi: 10.1002/ctm2.70787 (PMC13521806; doi:10.1002/ctm2.70787)
Supplement: Supplementary file 2 [file CTM2-16-e70787-s002.docx]

**Table S2. Antibodies used in the experiments.**

| **Antibody** | **Company** | **No. of Catalogue** | **WB** | **IHC** | **IF** |
| --- | --- | --- | --- | --- | --- |
| PRAME | Abcam, USA | ab219650 | 1:1000 | 1:100 |  |
| PRAME | Cell Signaling Technology, USA | 56426T | 1:1000 |  |  |
| PRAME | Proteintech, China | 68097-1-Ig | 1:10000 |  | 1:800 |
| RARα | Proteintech, China | 10331-1-AP | 1:1000 | 1:100 | 1:200 |
| Cullin2 | Abcam, USA | ab166917 | 1:1000 |  |  |
| RBX1 | Abcam, USA | ab133565 | 1:5000 |  |  |
| ELOB | Abcam, USA | ab151743 | 1:1000 |  |  |
| Ubiquitin | Proteintech, China | 80992-1-RR | 1:50000 |  |  |
| MMP2 | Proteintech, China | 66366-1-Ig | 1:1000 |  |  |
| GAPDH | Proteintech, China | 60004-1-Ig | 1:50000 |  |  |
| IgG  E-Cadherin  N-Cadherin  Twist1  Vimentin  K48-linkage Specific Polyubiquitin  K63-linkage Specific Polyubiquitin | Proteintech, China  Cell Signaling Technology, USA  Cell Signaling Technology, USA  Cell Signaling Technology, USA Cell Signaling Technology, USA  Cell Signaling Technology, USA  Cell Signaling Technology, USA | 30000-0-AP  3195T  14215T  90445T  5741T  8081T  5621T | 1:1000  1:1000  1:1000  1:1000  1:1000  1:1000  1:1000 |  |  |

Abbreviations: WB: Western blot; IHC: Immunohistochemistry; IF: Immunofluorescence.
